# Characterizing the heterogeneity of clinician practice use in community mental health using latent profile analysis

**DOI:** 10.1186/s12888-019-2234-0

**Published:** 2019-08-23

**Authors:** Emily M. Becker-Haimes, Viktor Lushin, Torrey A. Creed, Rinad S. Beidas

**Affiliations:** 1 Department of Psychiatry, University of Pennsylvania Perelman School of Medicine, 3535 Market Street, 3rd floor, Philadelphia, PA 19104 USA; 2 Hall Mercer Community Mental Health, Philadelphia, PA USA; 3 Department of Medical Ethics and Health Policy, University of Perelman School of Medicine, Philadelphia, USA; 4 Penn Implementation Science Center at the Leonard Davis Institute of Health Economics, Philadelphia, USA

**Keywords:** Usual care, Implementation science, Youth mental health, Latent profile analysis

## Abstract

**Background:**

The behavioral health service provider population is highly heterogeneous. However, it is rarely treated as such within evidence-based practice implementation efforts. This study aimed to evaluate, as a proof of concept, the utility of latent profile analysis to identify distinct profiles of clinician practices in a large sample of youth-serving community mental health clinicians. This study also aimed to identify predictors of profile membership to inform implementation efforts.

**Methods:**

Participants were 484 practicing clinicians (79.4% female, 45.7% White, *M* age = 37.1 years). As part of a larger survey, clinicians reported on their use of cognitive, behavioral, family, and psychodynamic treatment techniques with a representative client on their caseload. Latent profile analysis was used to determine the presence of clinician practice profiles. Multilevel multinomial logistic regressions examined predictors of profile membership.

**Results:**

Latent profile analysis indicated a 4-profile solution best fit the data, with clinicians who: 1) used generally low levels of all examined techniques and preferred cognitive techniques (*Low Eclectics,* 16%), 2) delivered moderate levels of all techniques (*Moderate Eclectics,* 53%), 3) demonstrated preference for use of family techniques (*Family Preferred*, 11%), and 4) used high levels of all techniques (*Super Users,* 20%). Clinician discipline (e.g., social work), education, and years of experience predicted profile membership.

**Conclusions:**

Findings from this proof of concept study underscore the utility of latent profile analysis to characterize the complex and heterogeneous makeup of community mental health. Results extend prior work highlighting the eclectic nature of community mental health practice. Predictor analyses underscore the important influence of clinician background characteristics on practice use.

## Background

Despite the availability of effective treatments for psychiatric disorders, most youth who receive treatment in community mental health systems do not receive evidence-based care [1, 2]. Recent efforts to increase the quality of care in community settings have focused on disseminating and implementing evidence-based practices (EBPs; [3]). However, evaluating the successes of these efforts by characterizing how clinicians use EBPs within community care has proved challenging [4–6]. Multiple studies conducted across the United States suggest that clinicians use techniques from different theoretical treatment models (e.g., cognitive behavioral therapy [CBT], family therapy, psychodynamic therapy) that have varying levels of empirical support for their efficacy in an eclectic fashion [7], and treatment often fails to lead to meaningful symptom reduction [8, 9]. Thus, while clinicians may indeed be delivering treatment techniques consistent with evidence, these are often concomitantly applied alongside techniques with less empirical support (e.g., [10]). Examination of practice *profiles* that can better characterize the range of techniques clinicians employ across theoretical treatment models*,* rather than examining the extent to which providers use techniques from individual treatments in isolation (e.g., behavioral techniques), may better elucidate how clinicians use various treatment techniques within community settings.

Effective characterization of clinician practice use is important both for evaluating the success of ongoing implementation efforts to increase clinician use of EBPs and for informing future implementation efforts. To date, characterizations of clinician treatment practices have relied on variable-centered approaches, or analyses that delineate relationships between variables (i.e., predicting outcomes, such as examining clinician attitudes as predictors of use of a single set of treatment techniques, such as CBT). These approaches assume homogeneity within the population of interest. However, this assumption is at odds with the makeup of the behavioral health service provider population which is heterogeneous in nature [11] and consists of individuals with diverse educational backgrounds that place variable emphasis on the use of treatment techniques consistent with EBP (e.g., social work, marriage and family therapy, clinical psychology; [12, 13]).

Person-centered analytic approaches, designed to identify subgroups of individuals within a larger sample using data-driven methods [14], offer an approach to understand the heterogeneity of clinician practice patterns in community mental health. Identifying the existence of clinician subgroups as well as identifying clinician characteristics predictive of subgroup membership (e.g., clinician participation in system-sponsored EBP implementation efforts, clinician discipline) would suggest the need to tailor implementation strategies as a function of clinician characteristics in addition to tailoring for specific organizational settings, as has been previously proposed [15]. Such work has the potential to help improve quality of care and outcomes of services in the public mental health system.

The objective of this proof-of-concept study is to evaluate the utility of latent profile analysis (LPA), a person-centered analytic approach, to characterize clinician practice patterns in one large public community mental health system, the City of Philadelphia, which has been actively implementing EBPs over the past decade. Prior work has demonstrated that an LPA approach successfully identified four distinct subgroups of clinicians as it pertains to standardized assessment practices [16]; however, to our knowledge, no work to date has applied this technique to identify profiles of clinician use of treatment practices. Using a large dataset of self-reported clinician treatment practices with youth, we examined whether distinct profiles of clinician practices were identifiable.

We hypothesized that, given the heterogeneity of the behavioral health workforce, an LPA approach would identify more than one distinct cluster of clinician practice profiles. While we considered the LPA to be exploratory in nature with respect to the anticipated number and nature of profiles, we did expect at least two distinct profile types to emerge: one of clinicians demonstrating largely eclectic practices, and others demonstrating preferences for specific sets of treatment techniques relative to others (e.g., clinicians using primarily cognitive and/or behavioral techniques relative to family and/or psychodynamic techniques). Beginning in 2007, the City of Philadelphia supported formal evidence-based practice implementation efforts focused on increasing clinician use of EBPs; efforts focused on youth mental health were primarily cognitive-behavioral in nature [17, 18]. Thus, we anticipated that while at least one practice profile would be consistent with an eclectic practice profile (i.e., use of techniques drawn equally from a range of treatment models), we expected to identify a subgroup of clinicians who demonstrate higher use of cognitive and/or behavioral techniques relative to other examined strategies.

In addition to identifying the existence of distinct profiles, we were also interested in identifying predictors of profile membership, for two reasons. First, profiles varying systematically by theorized predictors would provide preliminary support for the validation of identified profiles [19]. Second, identifying predictors of profile membership is important for informing *how* and *for whom* implementation strategies may need to be tailored with respect to clinician characteristics. Thus, we were primarily interested in examining clinician demographic and clinician training background characteristics as predictors of profile membership. Predictors included: professional discipline (social work, marriage and family, counseling), highest degree (master’s, doctoral, intern), licensure status, years of experience, and formal EBP implementation effort participation. These predictors were chosen because they are observable clinician characteristics that could facilitate tailoring of implementation strategies in future implementation efforts. While exact hypotheses for predictor analyses cannot be made without first establishing the existence of profiles, we did expect that clinicians who had participated in one or more of the City-sponsored CBT implementation initiatives would be more likely to belong to a profile(s) demonstrating higher use of cognitive and/or behavioral techniques relative to other examined techniques, assuming such a profile should emerge in the LPA. Other predictors were exploratory in nature.

## Methods

### Participants and procedures

Data were collected as part of a larger study examining the impact of a system transformation over the course of 5 years within the community mental health system in Philadelphia [20]. This study constituted secondary data analysis of this longitudinal dataset. All procedures were approved by the City of Philadelphia and University of Pennsylvania institutional review board as minimal risk to participants (Protocol Numbers 2012–41 and 816619, respectively). The City of Philadelphia served as the IRB of record and approved this study on October 2nd, 2012. All procedures performed were in accordance with the ethical standards of the 1964 Helsinki declaration and its later amendments. Written informed consent was obtained for all study participants.

Participants were 484 community mental health clinicians in the city of Philadelphia working across 31 agencies over 5 years (2013–2017). These agencies were purposively selected because they collectively served approximately 80% of youth receiving mental health services in the City of Philadelphia. During this five-year period, the Department of Behavioral Health and Intellectual Disability Services (DBHIDS) in Philadelphia supported five EBP implementation initiatives (cognitive therapy, prolonged exposure, trauma-focused cognitive behavioral therapy, dialectical behavior therapy, and parent-child interaction therapy; see [21] for a detailed overview). Survey data were collected from practicing clinicians in these 31 agencies at three waves of data collection over 5 years (Year 1, Year 3, and Year 5). At each time-point, clinicians consented to participate in the study and completed a series of questionnaires during a 1-time, 2-h meeting at each organization; 110 clinicians participated in 2 waves of data collection, 16 participated in all 3 waves. Given the low rate of clinicians who contributed data at multiple time points (22%), we used data from all clinicians’ initial participation timepoint to provide the broadest cross-sectional snapshot of practice use, consistent with prior analyses with this dataset examining predictors of specific clinician evidence-based practices [22].

A total of 499 unique clinicians across 31 clinical organizations participated across the 5 years (Year 1 clinician *n* = 130, organization *n* = 22; Year3 clinician *n* = 247, organization *n* = 28; Year 5 clinician *n* = 247, organization *n* = 25); 15 clinicians did not report on their practice use, yielding a final sample of 484. An average of 15.6 clinicians per agency participated (*SD* = 11.0, Range = 3–52); on average, this represented a participation rate of 67% for clinicians across the 31 organizations over the 3 waves of data collection.

### Measures

#### Clinician use of treatment techniques

**Fig. 1:**
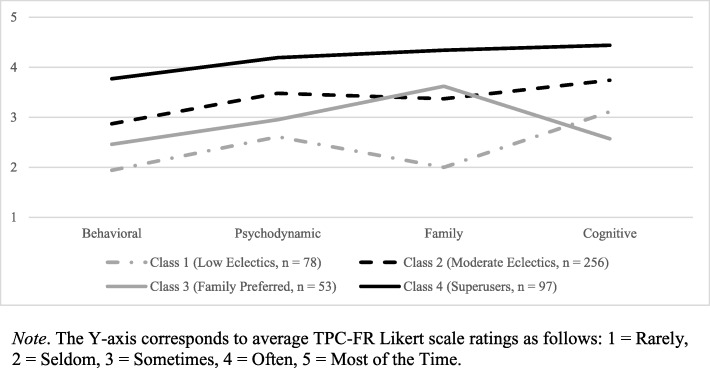
Treatment techniques endorsed for each of the 4 latent profiles identified. *Note*. The Y-axis corresponds to average TPC-FR Likert scale ratings as follows: 1 = Rarely, 2 = Seldom, 3 = Sometimes, 4 = Often, 5 = Most of the Time

Clinician treatment techniques were indexed via the Therapy Procedures Checklist – Family Revised (TPC-FR), a self-report measure of clinician use of treatment techniques with youth [23, 24]. The TPC-FR assesses clinician use of cognitive, behavioral, family, and psychodynamic techniques. In this study, clinicians were asked to select a representative client on their caseload and indicate the extent to which they used the 62 techniques on the TPC-FR with that client over the course of that client’s treatment. Items are rated on a 5-point Likert scale that ranges from 1 (*Rarely Use*) to 5 (*Use Most of the Time;* see Fig. 1 for anchor descriptions of the full Likert scale).

Of note, traditional methods of monitoring and/or indexing clinician practice use, such as adherence checklists or other standardized fidelity measurements [25] are derived from efficacy trials where all clinicians may deliver a circumscribed intervention (e.g., treatment manual) for specific diagnostic presentations. While such an approach is ideal for monitoring treatment integrity and standardization in randomized controlled trials, this approach presents challenges when applied to community settings. Multiple studies demonstrate treatment techniques used by clinicians in community settings are drawn from various treatment models (e.g., family systems therapy, cognitive-behavioral therapy, psychodynamic therapy; [7]). To our knowledge, the TPC-FR represents the only published self-report instrument of therapist practices that spans across multiple treatment protocols and theoretical orientations, making it a conceptually appropriate dependent measure for this proof-of-concept study. Prior work suggests that the TPC-FR demonstrates adequate psychometric properties, including test-retest reliability and sensitivity to within-therapist changes in technique use [23, 24]. Prior confirmatory factor analysis indicates the TPC-FR yields four subscales mapping onto the theoretical treatment models represented on the measure; Cognitive, Behavioral, Family, and Psychodynamic techniques [26]. All four subscales demonstrated good internal consistency in this sample (αs = .87–.93).

In addition to the 62 treatment techniques on the TPC-FR, clinicians also reported on demographic and clinical characteristics of this representative client (age, gender, ethnicity, primary diagnosis).

#### Clinician characteristic

Clinicians reported on demographic characteristics (age, gender, race/ethnicity) and clinical training background (e.g., degree obtained, discipline [social work, MFT, psychology, general mental health], years of experience, licensure status, and participation in one or more of the five formal EBP implementation efforts sponsored by DBHIDS). Participants verbally confirmed their understanding that the question about implementation participation referred to the formal year-long initiative training and consultation through DBHIDS. As noted above, EBP implementation efforts sponsored by DBHIDS for youth were primarily cognitive behavioral in nature.

### Analysis plan

We used latent profile analysis (LPA) to examine patterns of clinicians’ reported use of the four treatment models indexed by the TPC-FR subscale scores: cognitive, behavioral, psychodynamic, and family therapies (all continuous variables). Consistent with recent recommendations, we did not include auxiliary variables in the LPA classification analyses [27]. Of note, we considered taking a multilevel LPA approach [28, 29] due to the nested nature of our data; however, due to our relatively smaller sample size of organizations at Level 2 and some small sample sizes within clusters, we elected not to take this approach for the LPA classification; however, as detailed further below, subsequent analyses accounted for the nested structure of the data.

Exploratory model building began with a one-class model, continuing until fit indices suggested further classes did not improve fit. Consistent with guidelines [30], final model selection was determined through a combination of absolute fit indices (Bayesian information criterion [BIC], Akaike information criterion [AIC]) and comparative fit indices that determine whether the addition of subsequent classes will substantially improve model fit (Vuong-Lo-Mendell-Rubin likelihood ratio test [VLMR LRT], and the Lo-Mendell-Rubin Adjusted LRT test), along with parsimony, interpretability, and generalizability. All analyses were done in Mplus with robust (Huber-White) maximum likelihood algorithms. Following establishment of profiles, we first examined simple associations of clinician characteristics with profile membership via chi-square tests and analysis of variance. As noted above, we hypothesized that at least two profiles would emerge: one of clinicians demonstrating primarily eclectic practices, and others demonstrating preferences for specific sets of treatment techniques relative to others.

We then examined predictors of profile membership via two-level multinomial logistic regression to account for the nested data structure (clinicians within organizations), controlling for client characteristics (client age and diagnosis [internalizing, externalizing, or other]) at level one (as clinicians reported on one client each) identified as associated with clinician practice use [31]. Predictors were considered exploratory. Finally, due to differences in findings between initial analysis of variance and multilevel multinomial logistic regressions, we conducted post-hoc analyses to examine whether predictors varied as a function of organization.

## Results

Clinicians (*N* = 484) averaged 37.1 years of age (*SD* = 11.4), were primarily female (79.5%) and of diverse ethnic backgrounds (45.7% White; 27.5% African American, 6.2% Asian, 9.5% Other; 18.4% identified as Hispanic/Latino and 14 individuals did not report on race or ethnicity). Average years of experience across the sample was 8.5 (*SD* = 8.5). Highest degree obtained for most clinicians was a master’s (*n* = 385, 79.5%); 41 (8.5%) held a bachelor’s and 50 (10.3%) held a doctoral degree. Clinician discipline was also varied: 57 (11.8%) were social workers, 39 (8.1%) were marriage and family therapists (MFTs), 21 (4.3%) were psychologists; the remainder represented “other master’s level” providers; in this sample, this is comprised of primarily clinicians with backgrounds in mental health counseling (e.g., licensed professional counselors). Clinicians worked an average of 2.8 years (*SD* = 3.9) at their agency of employment; 43% (*n* = 207) of clinicians had participated in at least one DBHIDS sponsored EBP implementation initiatives. Analysis of variance and chi-squared tests indicated no significant differences in clinician demographics across the three waves (all *p*s > .05), with the exception that clinicians who participated at the final wave of data collection were slightly younger on average than therapists at the prior waves (*F* = 4.73,.*p* = .01).

### Profiles

Table 1 displays fit indices for 1–5 profile models. A 4-profile model yielded adequate model fit (AIC = 3838.325, BIC = 3934.513, Entropy = .78) and the Adjusted LRT indicated that a 5-profile model did not substantially improve fit beyond a 4-profile model (*p* > .05). In addition, while the 5-profile model marginally improved fit indices, this model produced two profiles that were nearly identical, with one profile that contained only 35 cases. Thus, the 4-profile solution was determined to be the most parsimonious profile solution.

**Table 1:** Comparative Model Fit of Latent Profile Solutions

Fit indices	Number of Profiles				
	1 Profile	2 Profiles	3 Profiles	4 Profiles	5 Profiles
Loglikelihood	− 2220.87	− 2008.40	− 1928.49	− 1896.16	− 1867.69
AIC	4457.73	4042.79	3892.97	3838.33	3791.38
BIC	4491.19	4097.16	3968.25	3934.51	3908.48
Entropy	–	0.70	0.78	0.78	0.79
Adjusted LRT	–	*p* < 0.01	*p* < 0.01	*p* = 0.05	*p* > 0.05

All analyses were done in Mplus with robust (Huber-White) maximum likelihood algorithms

Figure 1 illustrates the practice patterns that emerged in the 4-profile model and Table 2 shows the average scores for each set of treatment techniques within each profile. Subscale means within each profile were inspected to identify appropriate profile names. Profile 1, termed “Low Eclectics” was characterized by low levels of all four treatment models, with a slight preference for cognitive techniques over family techniques (*n* = 78, 16%). Profile 2 characterized the largest portion of the sample (*n* = 256, 53%). Termed “Moderate Eclectics”, these clinicians demonstrated moderate use of all four treatment models. Profile 3 represented the smallest portion of clinicians (*n* = 53, 11%) and was characterized as the “Family Preferred” profile; these clinicians also exhibited eclectic technique use, but demonstrated preference for family techniques over cognitive techniques. Finally, profile 4, termed the “Super Users” (*n* = 97, 20%), demonstrated high use of all treatment models.

**Table 2:** Descriptive statistics for treatment techniques and independent variables across profiles

	Low Eclectics *n* = 78	Moderate Eclectics *n* = 256	Family Preferred *n* = 53	Super Users *n* = 97	Test Statistic ^a^	*p*
Treatment Techniques						
Behavioral, *M* (SD)	1.94 (0.62)	2.86 (0.59)	2.46 (0.68)	3.77 (0.58)	*F*(3,480) = 141.12	<.01
Psychodynamic, *M* (SD)	2.61 (2.63)	3.48 (0.38)	2.95 (0.50)	4.19 (0.37)	*F*(3,480) = 211.45	<.01
Family, *M* (SD)	2.00 (0.49)	3.37 (0.60)	3.62 (0.55)	4.34 (0.37)	*F*(3,480) = 278.12	<.01
Cognitive, *M* (SD)	3.11 (0.58)	3.74 (0.40)	2.57 (0.44)	4.44 (0.31)	*F*(3,480) = 275.20	<.01
Clinician Characteristics						
Years of experience, *M* (SD)	6.59 (6.48)	8.47 (8.08)	4.48 (4.46)	12.32 (10.70)	*F*(3,469) = 12.32	<.01
Discipline ^b^						
Social Work	12 (16%)	36 (14%)	4 (8%)	6 (6%)	*Χ*^*2*^(3) = 5.83	.12
Marriage and Family Therapist	1 (1.3%)	19 (7%)	9 (17%)	10 (11%)	*Χ*^*2*^(3) = 11.51	<.01
Other Master’s (e.g., counselor)	49 (10%)	156 (32%)	30 (6%)	65 (13%)	*Χ*^*2*^(3) = 1.79	.62
EBP Initiatives Participation:					*Χ*^*2*^(6) = 5.85	.44
None	44 (57%)	144 (57%)	30 (60%)	50 (53%)		
One initiative	22 (28%)	71 (28)	14 (28%)	21 (22%)		
Two or more initiatives	11 (14%)	39 (15%)	6 (12%)	23 (24%)		
Highest Degree Obtained ^c^					*Χ*^*2*^(1) =13.72	<.01
Master’s	60 (78%)	204 (80%)	43 (86%)	78 (81%)		
Doctoral	13 (17%)	20 (8%)	0 (0%)	17 (18%)		
Professional Status						
Clinical Intern, *n* (%)	7 (9%)	29 (12%)	10 (19%)	3 (3%)	*Χ*^*2*^(1) = 10.44	.02
Clinically Licensed, *n* (%)	15 (19%)	59 (23%)	8 (16%)	13 (14%)	*Χ*^*2*^(3) = 4.54	.21
Client Characteristics						
Primary Diagnosis					*Χ*^*2*^(6) = 22.27	<.01
Externalizing disorder	34 (45%)	154 (61%)	30 (58%)	61 (69%)		
Internalizing disorder	33 (44%)	68 (27%)	9 (17%)	19 (22%)		
Other disorder (e.g., autism)	8 (10%)	29 (12%)	13 (25%)	8 (9%)		
Client Age	11.92 (3.60)	10.81 (3.34)	8.79 (3.43)	11.52 (3.34)	*F*(3,476) = 10.26	<.01

^a^ Analysis of variance conducted for continuous variables, chi-squared tests conducted for categorical variables

^b^ Due to small sample sizes for clinicians who identified their discipline as psychologists (*n* = 20) and psychiatrists (*n* = 1), these were not examined

^c^ Excludes individuals who reported their highest degree as a bachelor’s degree, as this overlapped with clinical intern status

Table 2 displays mean values for predictors (clinician characteristics) and covariates (client characteristics) of interest across the four classes and results of univariate chi-squared tests and analysis of variance. Simple associations indicated that clinicians with the most years of experience were in the Super User profile, MFTs clustered within the Family Preferred profile, interns were less likely to be in the Super User profile, and those with doctoral degrees were more likely to be in either the Low Eclectic or Super User profile. Client diagnosis and age were also associated with profile membership, suggesting these variables be controlled for in regression analyses.

Multilevel multinomial logistic regressions then examined predictors of profile membership to account for the nested data structure and to control for client characteristics (see Table 3). Given the large proportion of clinicians that fell into the Moderate Eclectics profile, which was also the profile most consistent with prior descriptions of usual care [7], all presented odds ratios (*OR*s) reflect the odds of profile membership relative to membership in the Moderate Eclectics profile. Overall, results were consistent with the univariate associations, with some differences. Specifically, relative to clinicians in the Moderate Eclectics profile, clinicians with more years of experience had a lower likelihood of belonging to the Family Preferred profile (*OR* = .91, [95% CI = .87, .95]) and greater likelihood of falling into the Super Users profile (*OR* = 1.03 [95% CI =1.01, 1.06]). Social Workers were less likely to be in the Super Users profile (*OR* = .46 [95% CI = .22, .97]). Clinicians with doctoral degrees were both more likely to belong to the Low Eclectics profile (*OR* = 2.52 [95% CI =1.05, 6.07]) and the Super User profile (*OR* = 2.10 [95% CI =1.22, 3.63]). Intern clinicians (*OR* = .34 [95% CI = .1, .83]) were less likely to belong to the Super User profile relative to non-intern clinicians. In addition, clinicians who were licensed (*OR* = .51 [95% CI = .35, .75]) were also less likely to belong to the Super User profile relative to unlicensed clinicians (i.e., the professional status of the Super User profile was primarily master’s level, unlicensed clinicians). Participation in one or more EBP implementation initiatives was not associated with profile membership (all *p*s > .05). Contrary to univariate analysis, being an MFT clinician was not related to profile membership (*OR* = .91 [95% CI = .77, 2.26]).

**Table 3:** Multinomial logistic regression analyses predicting profile membership

	Moderate Eclectics vs. Low Eclectics *OR* [95% CI] ^a^	Moderate Eclectics vs. Family Preferred *OR* [95% CI] ^a^	Moderate Eclectics vs. Super Users *OR* [95% CI] ^a^
Clinician Characteristics			
Years of experience	0.95 [0.89–1.00]	0.91* [0.87–0.95]	1.03* [1.01–1.06]
Discipline			
Social Work	0.92 [0.48–1.76]	0.62 [0.20–2.01]	0.46* [0.22–0.97]
Marriage and Family Therapist	0.17 [0.02–1.21]	0.91 [0.77–2.26]	0.88 [0.78–1.61]
Other Master’s (e.g., counselor)	1.23 [0.76–2.00]	0.84 [0.50–1.42]	1.20 [0.75–1.94]
EBP Initiatives Participation ^c^			
Zero vs. One initiative	0.85 [0.53–1.37]	1.00 [0.59–1.70]	0.85 [0.56–1.28]
Zero vs Two or more initiatives	0.90 [0.44–1.83]	0.94 [0.34–2.58]	1.62 [0.91–2.86]
One vs. Two or more initiatives	1.21 [0.75–1.96]	0.74 [0.41–1.31]	1.12 [0.73–1.72]
Highest Degree Obtained			
Doctoral Degree vs. Master’s	2.52* [1.05–6.07]	-- ^b^	2.10* [1.22–3.63]
Professional Status			
Clinical Intern	0.61 [0.33–1.17]	1.17 [0.46–2.96]	0.34* [0.1–0.83]
Clinically Licensed	0.75 [0.45–1.24]	0.88 [0.39–1.98]	0.51* [0.35–0.75]

*Note.* *p < .05. ** *p* < .01. *** *p* < .001

^a^ Odds Ratios reflect the odds associated with being in the low eclectic, family preferred, or super user profiles relative to the moderate eclectics, controlling for client characteristics (client age and primary diagnosis)

^b^ Profile does not have sufficient cases on specified variable

^c^ We also examined initiative as a dichotomous yes/no variable and results were similarly non-significant

### Post-hoc analyses

Given differences between univariate and multi-level analyses, we calculated post-hoc intraclass correlation coefficients (ICCs) to examine whether predictors varied as a function of organization; significant clustering of clinician characteristics within agencies may potentially explain these differences. Continuous variable ICCs reflect the proportion of variance attributable to organizations; categorical variable ICCs reflect the proportion of the probability of belonging to one category attributable to organizations. Significant variability in clinician backgrounds was attributable to the organizational level for all predictors, suggesting clinicians with similar clinical educational and training backgrounds clustered within organizations: years of experience ICC = .12, social worker ICC = .40, MFT ICC = .39, other master’s ICC = .20, intern ICC = .56, licensure status ICC = .17, and EBP implementation initiative participation ICC = .18.

## Discussion

Results of this LPA underscored the variability of community mental health practices and suggested that clinician practice patterns fall into one of four groups: 1) *Low Eclectics,* or clinicians who generally used low levels of all examined techniques and preferred cognitive techniques 2) *Moderate Eclectics,* or clinicians who delivered moderate levels of all, 3) *Family Preferred,* or clinicians who demonstrated preference for use of family therapy techniques, and 4) *Super Users,* or those clinicians who reported using high levels of all techniques. To our knowledge, this is the first study to apply person-centered analytic strategies to characterize clinician treatment practices with youth, providing proof of concept of the potential for this approach to characterize the heterogeneity of clinicians that comprise community care and their practice patterns. Findings supported the notion that nearly all clinicians employ an eclectic approach to treatment in community mental health settings, consistent with prior work (e.g., [7]). However, this eclecticism can be further characterized into four heterogeneous subgroups that demonstrate unique practice patterns that are differentiated by clinician background characteristics. While the use of self-reported practice data to some extent limits conclusions that can be drawn from this study, findings underscore the utility of the LPA approach; examining clinician use of cognitive strategies in isolation (i.e., treating various treatment models as orthogonal constructs) would obscure the fact that clinicians who reported the highest use of CBT also reported the highest use of psychodynamic techniques. The LPA approach allowed for simultaneous examination of treatments to capture the co-varying and eclectic nature of community mental health treatment.

Importantly, the clear differentiation across practice profiles as a function of clinician discipline and educational attainment highlights the potentially critical role pre-service (i.e., graduate) education and training plays in treatment delivery. Specifically, social workers were most likely to have a practice characterized by a moderate use of all four groups of treatment techniques, whereas MFT clinicians demonstrated some preference for family therapy techniques over other examined treatment models (although this latter finding was not significant once the nested data structure was accounted for in analysis). This makes conceptual sense; social work programs take a more holistic, ecological systems approach to practice [32], whereas MFT programs place emphasis on family systems techniques. In contrast, doctoral-trained providers were less likely to belong to the Moderate Eclectics profile. Coupled with the fact that participation in one or more EBP implementation initiatives was not associated with profile membership, results suggest that providers may rely on using practices learned in their graduate training, regardless of continuing education efforts. This is consistent with broader work in the medical field suggesting that physicians continue to practice medicine consistent with what is learned in their graduate medical education, regardless of ongoing continuing medical education [33].

While implementation initiative participation itself was not associated with profile membership, prior work has found that clinicians with more years of experience have participated in a greater number of EBP implementation efforts [34], suggesting that training in initiatives likely interacts with prior training and experience. Clinicians with more years of experience were most likely to have a practice pattern characterized by a high level of use of all four treatment models (i.e., the Super Users group), suggesting that while those clinicians with more experience reported the highest use of treatment techniques supported by evidence (e.g., cognitive and behavioral techniques), these clinicians also reported the highest rates of treatment techniques with the least empirical support (i.e., psychodynamic techniques). Given the number of cognitive-behavioral implementation initiatives that have occurred within the Philadelphia system over the past decade [21] this suggests that providers who are working in the system longer and participate in more training initiatives, may integrate techniques they learn via initiatives alongside those they were using prior to the implementation effort (e.g., those learned in graduate training), rather than de-implementing such techniques. This could also reflect social desirability and a belief that endorsing more practice use is best-practice [35].

Taken together, findings highlight the importance of considering the impact a clinician’s graduate discipline has on implementation outcomes. To date, implementation efforts have largely targeted post-service entry providers [1, 36], with little work addressing the varied exposure to training providers receive in their graduate training [11, 12, 37]. Results stress the need for a greater focus on understanding: 1) how a clinician’s initial training and education influences their practices over the course of their career, 2) how initial training interacts with ongoing implementation efforts with respect to clinician practice (e.g., whether and how providers layer new techniques on top of what they previously learned) as extensive work in both social psychology demonstrates the long-lasting impact of initial messages and training (e.g., [38]), 3) how graduate (pre-service) education can best prepare clinicians for the demands of delivering EBPs in the public mental health system, and 4) how to encourage de-implementation of practices with less empirical support for their use among practicing clinicians.

Results did not support the hypothesis that there are subpopulations of clinicians that use more “pure” treatment techniques (e.g., primarily cognitive and/or behavioral techniques or primarily psychodynamic techniques). In other words, no profile emerged that resembled what one might hypothesize clinician practice would look within the context of a randomized controlled trial (RCT). For example, a clinician delivering care in a CBT treatment arm of an RCT’s practice profile might show high intensity of cognitive and behavioral techniques with relatively minimal use of family or psychodynamic techniques. Furthermore, while many providers in the Moderate Eclectic and Super User profiles reported high rates of treatment techniques consistent with the evidence-base, (which is promising), they concomitantly reported high rates of treatment techniques with less empirical support (i.e., a mix of prescribed [EBP] and proscribed [non-EBP] treatment techniques, indicating little treatment differentiation; [39]). One can think of these practice profiles as representing “off-label,” or non-traditional use of EBPs. Providers may be using a variety of techniques to address the oftentimes more complex needs of youth seeking care through the community mental health system relative to youth treated in RCTs [40, 41]. However, it will be critical for future research in this area to link practice data such as that examined in this study with client outcome data to better understand how an eclectic approach that includes a high-level of EBPs differentially impacts client outcomes relative to eclectic treatment techniques delivered at a lower-intensity.

While this study primarily examined clinician characteristics influencing practice use, post-hoc analyses also demonstrated clustering of similar clinicians (e.g., those with similar training, disciplines) at the organizational level. While prior work has suggested that there is substantial variation in clinician practice use attributable to organizational influences [42], pointing to the utility of organizational-level implementation strategies for increasing EBP use [43], this study extends this work and suggests that aggregation of similar clinician within agencies may partially account for organizational variability in clinicians’ practice use. This is consistent with the attraction-selection-attrition (ASA) hypothesis in organizational theory, which posits a bidirectional influence of organizational and clinician characteristics through cycles in which individuals are attracted to certain organizations, organizations select to hire providers with an epistemological approach or background consistent with that organizations’ philosophy (e.g., a family-systems oriented agency prioritizes hiring MFT providers). This in turn contributes to a consolidation of organizational culture [44], reinforcing the clustering of practice use within organizations. EBP implementation efforts are also often rolled out at an agency level; agencies may be more likely to seek out EBP training consistent with their culture, although this was not tested in this study. Thus, while this study primarily highlights the heterogeneity of the mental health service population at the provider level and points to pre-service education as an important, yet understudied, implementation target, findings continue to support the need for multilevel implementation strategies when targeting the *existing* workforce. This extends prior work [15] by supporting the utility of tailoring implementation strategies to both clinicians *and* organizations.

### Limitations

The primary limitation of this proof-of-concept study is the use of a retrospective self-report measure to index clinician practice use. While self-report measures of clinician practice use represent a low-burden method of measurement and the TPC-FR specifically represents one of the only self-report instruments to assess clinician practices across treatment models, self-report data has known limitations [4, 45]. It will be important for further research to replicate these practice profiles using observational data. In addition, should sample sizes permit, accounting for organizational nesting in the profile classification via techniques such as multilevel LPA [27] will also be important for further research. Furthermore, we examined clinician-reported practices within an entire representative case on their caseload; results may differ if practice were indexed at the session level (e.g., individual sessions may include use of more targeted treatment techniques). Relatedly, client diagnosis and age were associated with clinician practices; we only had data on a single client per clinician and only broad information about clients’ clinical presentations (i.e., primary diagnosis). Recommendations for further research include replicating these findings with multiple clients per clinician to parse apart how clinician background characteristics interact with client demographic and clinical characteristics to predict practice patterns, as clinical practice that is tailored to individual presentations should pull for different treatment techniques [46].

In addition, while we intentionally selected observable clinician characteristics as predictors of interest in this study to facilitate their applicability to future implementation efforts, our predictors had limitations. Most notably, we were unable to examine how a clinician’s specific history with EBP training (e.g., in what specific city-sponsored initiatives clinicians), due to small sample sizes at this more granular level. There are also a host of variables across ecological levels that are theorized to influence clinician practice use (see [47] for an overview); a comprehensive review of all potential predictors of profile membership was considered outside the scope of this proof-of-concept study. Finally, licensure status and professional discipline emerged as variables associated with practice profiles. Licensure rates and compositional breakdown of providers (e.g., proportion of clinicians that are professional counselors versus social workers) vary by state. This work was conducted within a single system (Philadelphia), which may limit generalizability of findings. Replication in states with different regulatory environments will be important to understand generalizability of findings.

## Conclusions

This proof of concept study underscores the complex and heterogeneous makeup of community mental health, both in clinician characteristics and practice use. In addition, findings highlight the value of analytic techniques that allow for identifying subgroups of mental health clinicians; person-centered analyses may therefore be useful to employ as dependent variables in further research aimed at understanding implementation outcomes in mental health. Finally, results of predictor analyses suggest that more attention needs to be paid to intervening within clinicians’ formative training years (e.g., graduate training) to maximize the extent to which EBPs are used, as these early characteristics were some of the strongest predictors of profile membership to emerge in this study.

## Data Availability

The datasets used and analyzed during the current study are available from the corresponding author on reasonable request.

## Abbreviations

AIC: Akaike information criterion
ASA: Attraction-selection-attrition
BIC: Bayesian information criterion
CBT: Cognitive behavioral therapy
DBHIDS: Department of Behavioral Health and Intellectual Disability Services
EBP: Evidence-based practice
ICC: Intraclass correlation coefficient
LPA: Latent profile analysis
MFT: Marriage and family therapy
TPC-FR: Therapy procedures checklist – family revised

## References

[1] McHugh RK, Barlow DH (2010). The dissemination and implementation of evidence-based psychological treatments: a review of current efforts. Am Psychol.

[2] Kazdin AE (2017). Addressing the treatment gap: a key challenge for extending evidence-based psychosocial interventions. Behav Res Ther.

[3] Stirman SW, Buchhofer R, McLaulin JB, Evans AC, Beck AT (2009). Public-academic partnerships: the Beck initiative: a partnership to implement cognitive therapy in a community behavioral health system. Psychiatr Serv.

[4] Beidas RS, Maclean JC, Fishman J, Dorsey S, Schoenwald SK, Mandell DS (2016). A randomized trial to identify accurate and cost-effective fidelity measurement methods for cognitive-behavioral therapy: project FACTS study protocol. BMC Psychiatry.

[5] Garland AF, Hurlburt MS, Brookman-Frazee L, Taylor RM, Accurso EC (2010). Methodological challenges of characterizing usual care psychotherapeutic practice. Adm Policy Ment Hlth..

[6] Schoenwald SK, Garland AF, Chapman JE, Frazier SL, Sheidow AJ, Southam-Gerow MA (2011). Toward the effective and efficient measurement of implementation fidelity. Adm Policy Ment Hlth..

[7] Garland AF, Brookman-Frazee L, Hurlburt MS, Accurso EC, Zoffness RJ, Haine-Schlagel R (2010). Mental health care for children with disruptive behavior problems: a view inside therapists’ offices. Psychiatr Serv.

[8] Warren JS, Nelson PL, Mondragon SA, Baldwin SA, Burlingame GM (2010). Youth psychotherapy change trajectories and outcomes in usual care: community mental health versus managed care settings. J Consult Clin Psych..

[9] Smith AM, Jensen-Doss A (2017). Youth psychotherapy outcomes in usual care and predictors of outcome group membership. Psychol Serv.

[10] Beidas R, Skriner L, Adams D, Wolk CB, Stewart RE, Becker-Haimes E (2017). The relationship between consumer, clinician, and organizational characteristics and use of evidence-based and non-evidence-based therapy strategies in a public mental health system. Behav Res Ther.

[11] Becker-Haimes Emily M., Okamura Kelsie H., Baldwin Constance D., Wahesh Edward, Schmidt Christopher, Beidas Rinad S. (2019). Understanding the Landscape of Behavioral Health Pre-service Training to Inform Evidence-Based Intervention Implementation. Psychiatric Services.

[12] Weissman MM, Verdeli H, Gameroff MJ, Bledsoe SE, Betts K, Mufson L (2006). National survey of psychotherapy training in psychiatry, psychology, and social work. Arch Gen Psychiat.

[13] Boat T, Land M, Leslie L, Hoagwood K, Hawkins-Walsh E, McCabe M, et al. Workforce development to enhance the cognitive, affective, and behavioral health of children and youth: opportunities and barriers in child health care training. National Academy of Medicine. 2017;7. https://nam.edu/workforce-development-to-enhance-the-cognitive-affective-and-behavioral-health-of-children-and-youth-opportunities-and-barriers-in-child-health-care-training/.

[14] Muthén B, Muthén LK (2000). Integrating person-centered and variable-centered analyses: growth mixture modeling with latent trajectory classes. Alcohol Clin Exp Res.

[15] Powell BJ, Beidas RS, Lewis CC, Aarons GA, McMillen JC, Proctor EK (2017). Methods to improve the selection and tailoring of implementation strategies. J Behav Health Serv Res.

[16] Cook JR, Hausman EM, Jensen-Doss A, Hawley KM (2017). Assessment practices of child clinicians: results from a national survey. Assessment..

[17] Beidas RS, Adams DR, Kratz HE, Jackson K, Berkowitz S, Zinny A (2016). Lessons learned while building a trauma-informed public behavioral health system in the City of Philadelphia. Eval Program Plann.

[18] Creed TA, Frankel SA, German RE, Green KL, Jager-Hyman S, Taylor KP (2016). Implementation of transdiagnostic cognitive therapy in community behavioral health: the Beck Community initiative. J Consult Clin Psych..

[19] Lanza ST, Cooper BR (2016). Latent class analysis for developmental research. Child Dev Perspect.

[20] Beidas RS, Aarons G, Barg F, Evans A, Hadley T, Hoagwood K (2013). Policy to implementation: evidence-based practice in community mental health–study protocol. Implement Sci.

[21] Powell BJ, Beidas RS, Rubin RM, Stewart RE, Wolk CB, Matlin SL (2016). Applying the policy ecology framework to Philadelphia’s behavioral health transformation efforts. Adm Policy Ment Hlth..

[22] Becker-Haimes EM, Okamura KH, Wolk CB, Rubin R, Evans AC, Beidas RS (2017). Predictors of clinician use of exposure therapy in community mental health settings. J Anxiety Disord.

[23] Weersing VR, Weisz JR, Donenberg GR (2002). Development of the therapy procedures checklist: a therapist-report measure of technique use in child and adolescent treatment. J Clin Child Adolesc.

[24] Kolko DJ, Cohen JA, Mannarino AP, Baumann BL, Knudsen K (2009). Community treatment of child sexual abuse: a survey of practitioners in the National Child Traumatic Stress Network. Adm Policy Ment Hlth..

[25] McLeod BD, Southam-Gerow MA, Weisz JR (2009). Conceptual and methodological issues in treatment integrity measurement. School Psychol Rev.

[26] Do M-CW, E; Weersing, V. R.;. Examination of the psychometric properties of the Therapy Procedures Checklist– Family Revised. Association for Behavioral and Cognitive Therapies; National Harbor, 2012.

[27] Asparouhov T, Muthén B (2014). Auxiliary variables in mixture modeling: using the BCH method in Mplus to estimate a distal outcome model and an arbitrary secondary model. Mplus Web Notes.

[28] Henry KL, Muthén B (2010). Multilevel latent class analysis: an application of adolescent smoking typologies with individual and contextual predictors. Structural Equ Modeling.

[29] Mäkikangas Anne, Tolvanen Asko, Aunola Kaisa, Feldt Taru, Mauno Saija, Kinnunen Ulla (2018). Multilevel Latent Profile Analysis With Covariates. Organizational Research Methods.

[30] Nylund KL, Asparouhov T, Muthén BO (2007). Deciding on the number of classes in latent class analysis and growth mixture modeling: a Monte Carlo simulation study. Struct Equ Model..

[31] Benjamin Wolk C, Marcus SC, Weersing VR, Hawley KM, Evans AC, Hurford MO (2016). Therapist-and client-level predictors of use of therapy techniques during implementation in a large public mental health system. Psychiatr Serv.

[32] Gitterman A, Knight C (2013). Evidence-guided practice: integrating the science and art of social work. Fam Soc.

[33] Nissen SE (2015). Reforming the continuing medical education system. JAMA..

[34] Skriner LC, Wolk CB, Stewart RE, Adams DR, Rubin RM, Evans AC, et al. Therapist and organizational factors associated with participation in evidence-based practice initiatives in a large urban publicly-funded mental health system. J Behav Health Serv Res. 2018:1–13. 10.1007/s11414-017-9552-0. 10.1007/s11414-017-9552-0 PMC5654710 28439788

[35] Izmirian SC, Nakamura BJ (2016). Knowledge, attitudes, social desirability, and organizational characteristics in youth mental health services. J Behav Health Serv Res..

[36] Novins DK, Green AE, Legha RK, Aarons GA (2013). Dissemination and implementation of evidence-based practices for child and adolescent mental health: A systematic review. J Am Acad Child Psy.

[37] Bertram RM, Charnin LA, Kerns SE, Long AC (2015). Evidence-based practices in north American MSW curricula. Res Social Work Prac.

[38] Chan M-pS, Jones CR, Hall Jamieson K, Albarracín D (2017). Debunking: a meta-analysis of the psychological efficacy of messages countering misinformation. Psychol Sci.

[39] McLeod BD, Southam-Gerow MA, Tully CB, Rodriguez A, Smith MM (2013). Making a case for treatment integrity as a psychosocial treatment quality indicator for youth mental health care. Clin Psychol Sci Pr.

[40] Chorpita BF, Bernstein A, Daleiden EL (2011). Empirically guided coordination of multiple evidence-based treatments: an illustration of relevance mapping in children's mental health services. J Consult Clin Psych.

[41] Southam-Gerow MA, Chorpita BF, Miller LM, Gleacher AA (2008). Are children with anxiety disorders privately referred to a university clinic like those referred from the public mental health system?. Adm Policy Ment Hlth..

[42] Beidas RS, Marcus S, Aarons GA, Hoagwood KE, Schoenwald S, Evans AC (2015). Predictors of community therapists’ use of therapy techniques in a large public mental health system. JAMA Pediatr.

[43] Williams NJ (2016). Multilevel mechanisms of implementation strategies in mental health: integrating theory, research, and practice. Adm Policy Ment Hlth.

[44] Schneider B, Goldstiein HW, Smith DB (1995). The ASA framework: an update. Pers Psychol.

[45] Hogue A, Dauber S, Lichvar E, Bobek M, Henderson CE (2015). Validity of therapist self-report ratings of fidelity to evidence-based practices for adolescent behavior problems: correspondence between therapists and observers. Adm Policy Ment Hlth..

[46] Ng MY, Weisz JR (2016). Annual research review: building a science of personalized intervention for youth mental health. J Child Psychol Psyc.

[47] Damschroder LJ, Aron DC, Keith RE, Kirsh SR, Alexander JA, Lowery JC (2009). Fostering implementation of health services research findings into practice: a consolidated framework for advancing implementation science. Implement Sci.

